# Ambiguity in the Presentation of Decellularized Tissue Composition: The Need for Standardized Approaches

**DOI:** 10.1111/aor.12838

**Published:** 2016-12-07

**Authors:** Arne A.N. Bruyneel, Carolyn A. Carr

**Affiliations:** ^1^ Department of Physiology, Anatomy and Genetics University of Oxford Oxford UK

**Keywords:** Tissue engineering—Decellularization—Normalization—Extracellular matrix, —Decellularization, —Normalization, —Extracellular matrix

## Abstract

Decellularization offers great potential to the field of tissue engineering, as this method gives rise to scaffold material with the native organ architecture by removing all cellular material and leaving much of the extracellular matrix (ECM) intact. However, many parameters may affect decellularization efficacy and ECM retention and, therefore, decellularization protocols need to be optimized for specific needs. This requires robust methods for comparison of decellularized tissue composition. Various representation methods are used in literature to express tissue composition (DNA, glycosaminoglycans, collagen, other ECM proteins, and growth factors). Here, we present and compare the various methods used and demonstrate that normalization to either dry or wet decellularized weight might be misleading and may overestimate true component retention. Moreover, the magnitude of the confounding effect is likely to be decellularization treatment dependent. As a result, we propose alternative comparison strategies: normalization to whole organ or to a unit of whole initial organ weight. We believe proper assessment of decellularized tissue composition is paramount for the successful comparison of different decellularization protocols and clinical translation.

Tissue engineering and biomaterial strategies might provide curative treatment for currently unmet clinical needs 1, 2. Decellularization offers great potential to the field of tissue engineering as it allows the generation of scaffold material with native organ structure and vasculature by washing out the cellular material 3, 4. Multiple physical, biological, and chemical means have been used to decellularize tissues with varying efficacy 5, 6, 7, 8. Detergents often form an essential part of most decellularization protocols and ionic detergents, such as sodium dodecyl sulfate (SDS), have the highest efficiency in removing cellular material 4. Decellularization requires total removal of DNA, but retention of collagen and glycosaminoglycans (GAGs) are also often reported 9, 10. Collagen is the major structural component of the ECM, and GAGs are polysaccharide ECM components that play a role in water retention and growth factor sequestering 11, 12.

Many parameters may affect decellularization efficacy and ECM retention and, therefore, decellularization protocols need to be optimized for specific needs. This requires robust methods for comparison of decellularized tissue composition. Accurate quantification by using sensitive detection techniques is essential, but the normalization strategy used might also affect the relative and absolute magnitudes of certain parameter values and their relative comparability, as both the numerator and the denominator might be affected by the treatment. We believe that the quantification of the GAG content might be most confounded by incorrect normalization, which is especially worrying because GAGs play important roles in the ECM from both a mechanical and biochemical point of view 11, 13.

To assess the range of normalization strategies reported in the literature, we ran a PubMed search on “decellularization,” “decellularized,” “decellularisation,” or “decellularised” using papers published between January 2014 and June 2015. All papers quantifying at least one of the components of interest (DNA, collagen, or GAG) were analyzed, including editorials (1%), reviews (14%), and experimental studies (72%, Fig. 1A). Measurement of protein and elastin was also recorded, but these were assessed in only 9 and 16 papers, respectively, and therefore are not discussed here. Almost half of the experimental studies did not perform any quantification of the retained ECM (48%), only presenting qualitative histology, while others only assessed DNA content (22%). For those papers that quantified multiple components (DNA, collagen, GAG, elastin, and protein), the majority used a consistent approach across all analyses (Fig. 1B, 41%), although some did not specify enough information to determine which method was used or whether it was used in a consistent manner (Fig. 1B, 6%). In some cases (Fig. 1B, 7%), different normalization strategies were used for different components.

**Figure 1:**
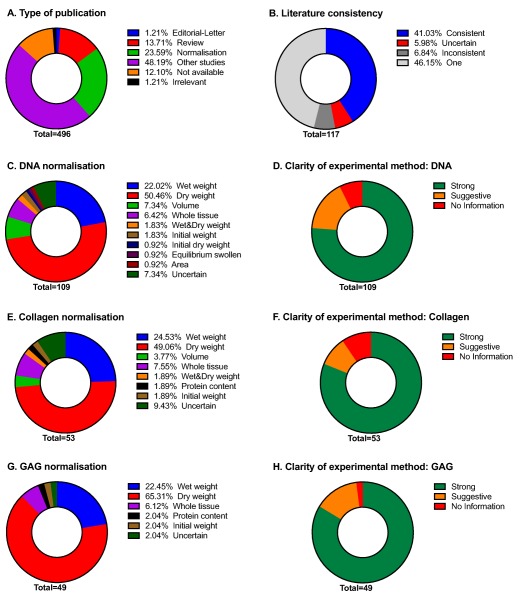
Literature review of normalization strategies. (A) Overview of the types of publications retrieved from PubMed, (B) within‐paper consistency of used normalization strategies, (C) distribution of strategies used for DNA content normalization, (D) clarity of methods describing DNA content normalization strategies, (E) distribution of strategies used for collagen content normalization, (F) clarity of methods describing collagen content normalization strategies, (G) distribution of strategies used for GAG content normalization, and (H) clarity of methods describing GAG content normalization strategies. [Color figure can be viewed at wileyonlinelibrary.com]

We were able to determine 11 different normalization strategies: (i) wet tissue weight, (ii) dry tissue weight, (iii) volume, (iv) whole tissue, (v) both wet and dry weight, (vi) protein content, (vii) initial tissue weight, (viii) initial dry weight, (ix) equilibrium swollen state, (x) area, and (xi) uncertain. In a significant proportion of papers, the methods section or the figure legend did not clearly describe the normalization (Fig. 1D,F,H), although the text suggested the most likely method. The classifier “uncertain” was only applied if there were multiple possible strategies. In part, the variety of normalization strategies reflects the diversity of research in which decellularized tissue is used, inter alia various organs, tissues, and cells cultured in vitro.

In general, normalization to wet or dry weight was the most common (Fig. 1C,E,G). Most papers reported reductions in DNA content. We observed that 65% of papers used normalization to dry weight for GAG quantification and 50% for collagen quantification. In most cases, authors observed comparable or reduced GAG and collagen content, although some papers reported counter‐intuitive increases in GAG and collagen content 14, 15, 16, 17, which most likely arose from inappropriate normalization. Papers using normalization to wet weight predominantly observed reductions or unchanged levels of GAG content. Very few papers normalized to whole‐organ weight but those that did observed comparable or reduced levels of GAG, depending on the harshness of the decellularization protocol 18. No papers using wet weight or whole‐tissue weight observed increases in GAG content.

Therefore, to optimize the decellularization successfully, appropriate comparison and analysis methods are paramount. In this study, we decellularized rat hearts with two detergents of markedly different efficiencies—SDS, an ionic detergent, or polyoxyethylene (10) tridecyl ether (POETE), a non‐ionic detergent—to demonstrate the effect of different methods for normalization and assess their confounding effect on quantitative component qualification.

## MATERIALS AND METHODS

### Animals

All procedures were carried out under an approved Home Office project license (number PPL 30/2755) in accordance with the Animals (Scientific Procedures) Act, 1986 (UK) (amended 2013) and approved by The University of Oxford Animal Ethics Review Committee. Male Sprague Dawley rats (Harlan UK; *n* = 4 per group, body weight 400–480 g) were kept under controlled conditions for temperature, humidity, and light, with environmental enrichment and with water and rodent chow available ad libitum. Animals were anesthetized by overdose of isofluorane or with sodium pentobarbital (200 mg/kg body weight, IP; Euthatal, Merial, UK) to allow tissue removal.

### Decellularization

Hearts were canulated via the ascending aorta and perfused briefly with Krebs buffer to wash out the blood. The hearts were decellularized by constant pressure perfusion (∼72 mm Hg) for about 18 h with detergent: 1% SDS in deionized water or 1% POETE in 50 mM Tris–HCl pH 7.4, washed for 5.5 h with deionized water, and subsequently fixed for histology or freeze clamped in liquid nitrogen for further analysis.

### Assessment of tissue structure and composition

For histology: hearts were fixed in 4% paraformaldehyde (PFA), embedded in paraffin, and sectioned. Sections were stained with hematoxylin and eosin (H&E) or Masson's trichrome (MT). For tissue composition: the tissue was crushed with a mortar and pestle under liquid nitrogen. Samples were weighed out and subsequently analyzed according to standard protocols. DNA were extracted with a DNA blood and tissue kit (Qiagen) and quantified using a Nanodrop spectrophotometer, according to the manufacturer's protocol. To determine collagen content, tissue was acid digested in 6 M HCl, dried, and the dry residue was dissolved. Hydroxyproline content was measured using Ehrlich's reagent 19. GAG content was measured with a Blyscan kit (Biocolor, Carrickfergus, County Antrim, UK), according to the supplied protocol.

### Normalization

Whole‐heart weights were recorded before and after decellularization. Frozen tissue was crushed under liquid nitrogen and a representative sample taken for dry‐weight determination by drying in an oven to a constant weight (48 h, 70°C).

Experiments were performed on wet tissue, thereby giving rise to results normalized to final wet weight. Normalization to initial wet weight used the following equation (for compound Q):

$$\mu g\;Q\;\text{per}\;\text{mg}\;\text{initial}\;\text{wet}\;\text{weight}=\frac{\mu g\;Q\;\text{per}\;\text{mg}\;\text{final}\;\text{wet}\;\text{weight}\;\times \;\text{total}\;\text{final}\;\text{heart}\;\text{wet}\;\text{weight}\;(\text{mg})\;}{\text{total}\;\text{initial}\;\text{heart}\;\text{weight}\;(\text{mg})}$$

For normalization to final dry weight:

$$\mu g\;Q\;\text{per}\;\text{mg}\;\text{of}\;\text{final}\;\text{dry}\;\text{weight}\;=\;\frac{\mu g\;Q\;\text{per}\;\text{mg}\;\text{final}\;\text{wet}\;\text{weight}}{\text{mg}\;\text{dry}\;\text{weight}\;\text{per}\;\text{mg}\;\text{final}\;\text{wet}\;\text{weight}}$$

Normalization to whole organ weight:

μg Q per whole organ=μg Q per mg final wet weight × total final heart weight (mg)

### Statistics

The data were analyzed using R or Prism Graphpad. Differences were considered significant when *P* ≤0.05: *P ≤0.05; **P ≤0.01, ***P ≤0.001; ****P ≤0.0001. Graphs represent data as mean ± SD. To test for significance, one‐way fixed‐effects ANOVA was used, provided the assumptions were met. Normality and homoscedasticity of the residuals were visually inspected, and more formally normality was tested using the Shapiro‐Wilk Normality Test. Homoscedasticity of the response variables were assessed using the Levene test. Conditional on rejecting a null effect in ANOVA, the Tukey Honest Significant Differences method was used for multiple comparisons. In case of heteroscedasticity, the Welch one‐way test was used, followed by Holm‐corrected multiple comparisons. In cases where the assumptions were violated for parametric tests, the Kruskal–Wallis Rank Sum Test was used.

## RESULTS

### Decellularization of isolated rat hearts

**Figure 2:**
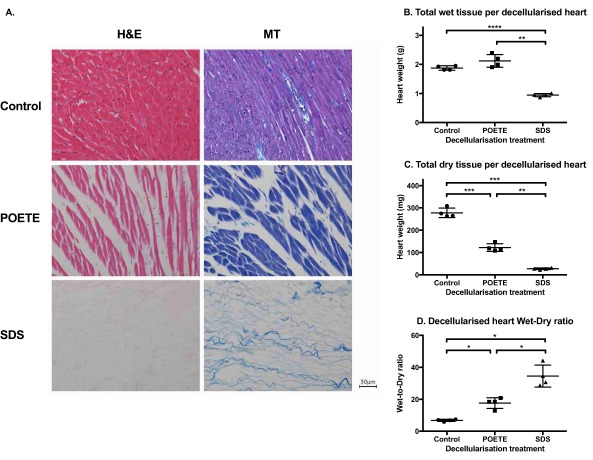
Histology of control and decellularized hearts and the contribution of water to the weight of decellularized tissue. Hearts were perfusion decellularized with either SDS or POETE. (A) Histology. The decellularized hearts were fixed in PFA, embedded in paraffin, sectioned, and stained with hematoxylin and eosin (H&E) or Masson's trichrome (MT). To determine the contribution of water to the weight of decellularized tissue, decellularized tissue samples were frozen in liquid nitrogen and crushed to powder, dried, and weighed out. (B) Total wet weight per decellularized heart, (C) total dry tissue per decellularized heart, and (D) wet‐to‐dry weight ratio. All data are presented as mean ± SD. *P ≤ 0.05; **P ≤ 0.01; ***P ≤ 0.001; ****P ≤ 0.0001. •, control; ▪ and ▲, POETE and SDS decellularization, respectively. [Color figure can be viewed at wileyonlinelibrary.com]

To study the confounding effect of normalization, hearts from rats of comparable body weight were decellularized with either SDS or POETE as detergent by constant pressure perfusion (*n* = 4 per group). SDS is an ionic detergent and has been reported to decellularize organs fully 3, 4. POETE, in contrast, belongs to the class of nonionic detergents and is ineffective at stripping tissue of cellular material. We deliberately selected detergents of variable efficacy as they are likely to differentially affect both numerator and denominator in the quantification of residual DNA, collagen, and GAG. Histological staining indicated that SDS treatment resulted in full decellularization, whereas POETE treatment only partly decellularized the tissue (Fig. 2).

### Dry matter content of decellularized tissue

Decellularization washes out cellular material from the tissue matrix and, as would be expected, total dry content decreased with decellularization. Similarly, total wet mass decreased in SDS‐treated samples compared to control and POETE. Moreover, the wet‐to‐dry ratio increased with decellularization efficacy: SDS > POETE > Control (Fig. 2), suggesting that during decellularization cellular material had been replaced by water. As a result, we postulate that different normalization strategies that relate content to either dry or final wet weight will not necessarily result in the same data interpretation.

### Analysis of retained material and comparison of normalization strategies

The collagen, DNA, and GAG content of the decellularized tissue were measured and we compared normalization to initial and final wet weight and final dry weight (Table 1). Both initial and final wet weights were compared because the water absorption is different in different decellularization protocols. In general, different values, relative differences, and distinct conclusions were reached depending on the normalization strategy used. Collagen content was maintained after decellularization when normalized to initial wet weight, whereas when normalized to final wet or dry weight, the collagen content was significantly higher in the SDS hearts. DNA content in SDS hearts was significantly reduced compared to control hearts for both initial and final wet weight. However, the extent of the reduction (Control vs. SDS) was different: showing a reduction to 5 or 10%, for initial and final wet weight, respectively. Strikingly, when normalized to dry weight, DNA content in POETE hearts was determined to be higher than in control or SDS hearts; whereas when normalized to wet after, DNA content was reduced and, when normalized to wet before, the reduction was not statistically significant. GAG content was significantly reduced by decellularization, with SDS hearts having almost undetectable levels, when normalized to initial or final weight. Comparable to the DNA results, the extent of the GAG content reduction was different depending on the normalization strategy: a reduction to 4 or 10%, for initial and final wet weight, respectively. When normalized to final dry weight, GAG content in SDS hearts was reduced to 50% compared to control, albeit not significantly, and increased in POETE hearts compared to control.

**Table 1:** Comparison of normalization strategies expressing tissue composition per unit of wet or dry weight or whole organ

	Wet before (µg/mg tissue)			Wet after (µg/mg tissue)			Dry after (µg/mg tissue)			Whole organ (mg/heart)		
	Control	POETE	SDS	Control	POETE	SDS	Control	POETE	SDS	Control	POETE	SDS
Collagen	6.3 ± 1.5	5.6 ± 0.4	5.0 ± 0.6	5.0 ± 0.7	4.8 ± 0.7	10.0 ± 1.1**** $$$$	33.9 ± 3.9	84.0 ± 6.6***	340.8 ± 49.5** $$	9.4 ± 1.2	10.1 ± 0.6	9.3 ± 0.8
DNA	1.3 ± 0.4	0.8 ± 0.1	0.06 ± 0.03* $$	1.0 ± 0.1	0.62 ± 0.04**	0.11 ± 0.05*** $$$$	7.0 ± 1.2	10.8 ± 1.4*	4.2 ± 2.4$	1.9 ± 0.3	1.3 ± 0.07*	0.11 ± 0.05** $$$$
GAG	1.2 ± 0.2	0.7 ± 0.02*	0.05 ± 0.04** $$$$	1.0 ± 0.1	0.54 ± 0.09***	0.10 ± 0.07**** $$$	6.7 ± 0.8	9.4 ± 1.2*	3.3 ± 2.6$	1.9 ± 0.2	1.1 ± 0.1**	0.09 ± 0.07*** $$$$

Hearts (*n* = 4 per group) were perfusion decellularized with either SDS or POETE. The samples were frozen in liquid nitrogen and crushed to powder, which was then analyzed for the presence of collagen, DNA, and GAG. All data are presented as mean ± SD, * or $ denotes P ≤0.05, ** or $$ P ≤0.01, *** or $$$ P ≤0.001 and **** or $$$$ P ≤0.0001 compared to control or POETE, respectively.

Alternatively, components can be expressed as the total amount in the whole organ (Table 1). For some tissues or organs, the abstraction total organ is not relevant (e.g., adipose tissue, skin, etc.), but for organs such as heart, lung, and kidney isolated from animals with roughly similar size, the measurement per organ may be appropriate and easier to visualize.

## DISCUSSION

Crapo et al. 4 proposed that the following minimal criteria should be used to assess efficacy of decellularization: <50 ng dsDNA per mg ECM dry weight, <200 bp DNA fragment length, and lack of visible nuclear material in tissue sections stained with 4′,6‐diamidino‐2‐phenylindole (DAPI) or H&E. Most tissues contain between 65 and 85% (by mass) water, although adipose tissue contains considerably less (10–30%). Fat accounts for 0–10% of most tissues and protein for 15–25% 20. After decellularization, the tissue will contain considerably less protein material, because cellular material is washed out, but the decellularized material still contains a considerable amount of water which is in cavities and/or bound by the material. We found that, although the total wet mass of hearts decellularized with SDS was significantly less than that of control or POETE‐treated hearts, the wet‐to‐dry ratio increased with the efficiency of decellularization (Fig. 2) as the decellularized tissue retained water more efficiently than the intact or partially decellularized tissue. In the literature, water‐binding ratios for collagen of about 20 g water per gram collagen sponge have been reported 21, 22. However, collagen sponges with GAGs (chondroitin sulphate) have significantly higher water binding capacity, depending on which cross‐linking method was utilized and, importantly, water‐binding capacity correlated positively with GAG content 21, 23. Therefore, materials that contain more GAGs will contain more water, and therefore might appear to have comparable GAG content per wet weight to a material that contains considerably less GAGs but also less water. Different decellularization protocols might extract various ECM constituents with different efficacies which would therefore affect water‐binding capacity and compromise normalization to final wet weight. Similarly, very strong decellularization treatments might appear to result in good retention, or even enrichment, of ECM components when expressed to dry weight, because total dry content is substantially different between groups. In our study, collagen, DNA, and GAG appeared to have been enriched by POETE decellularization, whereas SDS decellularization appeared to have caused a small, but not significant, decrease in DNA and GAG content but to have enriched the collagen content 10‐fold.

It was recently suggested that the FDA and CE are jeopardizing safety by allowing fast commercialization of decellularized tissue for clinical application in humans and that there is an urgent need for legislation to deal with quality control and safety of decellularized tissue 24. We believe the authors are rightly worried as we have demonstrated that comparisons based on final dry or wet weight, which are the most commonly used, could be misleading.

## CONCLUSION

Many parameters affect decellularization efficacy, and decellularization protocols need to be optimized for specific needs, for which good comparison methods are paramount. It is clear that proper assessment of ECM protein retention is paramount for the successful comparison of decellularization protocols and for safe clinical translation. We have shown here that the use of pretreatment tissue weight or whole‐organ weight provides the most appropriate normalization.

## Author Contributions

AB: concept/design, data analysis/interpretation, drafting and critical revision of article, and statistics; CC: concept/design, critical revision of article, approval of article, and funding secured.

## Conflict of Interest

None of the authors reports a conflict of interest.

## References

[1] Ott LM , Weatherly RA , Detamore MS. Overview of tracheal tissue engineering: clinical need drives the laboratory approach. Ann Biomed Eng 2011;39:2091–113. 21594727 10.1007/s10439-011-0318-1

[2] Fröhlich M , Grayson WL , Wan LQ , Marolt D , Drobnic M , Vunjak‐Novakovic G. Tissue engineered bone grafts: biological requirements, tissue culture and clinical relevance. Curr Stem Cell Res Ther 2008;3:254–64. 19075755 10.2174/157488808786733962 PMC2773298

[3] He M , Callanan A. Comparison of methods for whole‐organ decellularization in tissue engineering of bioartificial organs. Tissue Eng Part B Rev 2013;19:194–208. 23083305 10.1089/ten.teb.2012.0340 PMC3627431

[4] Crapo PM , Gilbert TW , Badylak SF. An overview of tissue and whole organ decellularization processes. Biomaterials 2011;32:3233–43. 21296410 10.1016/j.biomaterials.2011.01.057 PMC3084613

[5] Lin P , Chan WCW , Badylak SF , Bhatia SN. Assessing porcine liver‐derived biomatrix for hepatic tissue engineering. Tissue Eng 2004;10:1046–53. 15363162 10.1089/ten.2004.10.1046

[6] Guyette JP , Gilpin SE , Charest JM , Tapias LF , Ren X , Ott H. Perfusion decellularization of whole organs. Nat Protoc 2014;9:1451–68. 24874812 10.1038/nprot.2014.097

[7] Meyer SR , Chiu B , Churchill TA , Zhu L , Lakey JRT , Ross DB. Comparison of aortic valve allograft decellularization techniques in the rat. J Biomed Mater Res 2006;79:254–62. 10.1002/jbm.a.30777 16817222

[8] Brown BN , Freund JM , Han L , et al. Comparison of three methods for the derivation of a biologic scaffold composed of adipose tissue extracellular matrix. Tissue Eng Part C Methods 2011;17:411–21. 21043998 10.1089/ten.tec.2010.0342 PMC3065729

[9] Tsuchiya T , Balestrini JL , Mendez J , Calle EA , Zhao L , Niklason LE. Influence of pH on extracellular matrix preservation during lung decellularization. Tissue Eng Part C Methods 2014;20:1028–36. 24735501 10.1089/ten.tec.2013.0492 PMC4241865

[10] Wang Y , Bao J , Wu Q , et al. Method for perfusion decellularization of porcine whole liver and kidney for use as a scaffold for clinical‐scale bioengineering engrafts. Xenotransplantation 2015;22:48–61. 25291435 10.1111/xen.12141

[11] Raman R , Sasisekharan V , Sasisekharan R. Structural insights into biological roles of protein‐glycosaminoglycan interactions. Chem Biol 2005;12:267–77. 15797210 10.1016/j.chembiol.2004.11.020

[12] Frantz C , Stewart KM , Weaver VM. The extracellular matrix at a glance. J Cell Sci 2010;123:4195–200. 21123617 10.1242/jcs.023820 PMC2995612

[13] Lovekamp JJ , Simionescu DT , Mercuri JJ , Zubiate B , Sacks MS , Vyavahare NR. Stability and function of glycosaminoglycans in porcine bioprosthetic heart valves. Biomaterials 2006;27:1507–18. 16144707 10.1016/j.biomaterials.2005.08.003 PMC2262164

[14] Bühler NEM , Schulze‐Osthoff K , Königsrainer A , Schenk M. Controlled processing of a full‐sized porcine liver to a decellularized matrix in 24 h. J Biosci Bioeng 2014;119:609–13. 25468420 10.1016/j.jbiosc.2014.10.019

[15] Sabetkish S , Kajbafzadeh A‐M , Sabetkish N , et al. Whole‐organ tissue engineering: decellularization and recellularization of three‐dimensional matrix liver scaffolds. J Biomed Mater Res Part A 2015;103A:1498–1508. 10.1002/jbm.a.35291 25045886

[16] Hussein KH , Park KM , Ghim JH , Yang SR , Woo HM. Three dimensional culture of HepG2 liver cells on a rat decellularized liver matrix for pharmacological studies. J Biomed Mater Res Part B Appl Biomater 2016;104:263–73. 25726837 10.1002/jbm.b.33384

[17] Baptista PM , Siddiqui MM , Lozier G , Rodriguez SR , Atala A , Soker S. The use of whole organ decellularization for the generation of a vascularized liver organoid. Hepatology 2011;53:604–17. 21274881 10.1002/hep.24067

[18] Xing Q , Yates K , Tahtinen M , Shearier E , Qian Z , Zhao F. Decellularization of fibroblast cell sheets for natural extracellular matrix scaffold preparation. Tissue Eng Part C Methods 2015;21:77–87. 24866751 10.1089/ten.tec.2013.0666 PMC4291209

[19] Chiariello M , Ambrosio G , Cappelli‐Bigazzi M , Perrone‐Filardi P , Brigante F , Sifola C. A biochemical method for the quantitation of myocardial scarring after experimental coronary artery occlusion. J Mol Cell Cardiol 1986;18:283–90. 3083110 10.1016/s0022-2828(86)80410-2

[20] Woodard HQ , White DR. The composition of body tissues. Br J Radiol 1986;59:1209–18. 3801800 10.1259/0007-1285-59-708-1209

[21] Pieper JS , Oosterhof A , Dijkstra PJ , Veerkamp JH , van Kuppevelt TH. Preparation and characterization of porous crosslinked collagenous matrices containing bioavailable chondroitin sulphate. Biomaterials 1999;20:847–58. 10226711 10.1016/s0142-9612(98)00240-3

[22] Chvapil M. Collagen sponge: theory and practice of medical applications. J Biomed Mater Res 1977;11:721–41. 893491 10.1002/jbm.820110508

[23] van Susante JLC , Pieper JS , Buma P , et al. Linkage of chondroitin‐sulfate to type I collagen scaffolds stimulates the bioactivity of seeded chondrocytes in vitro. Biomaterials 2001;22:2359–69. 11511033 10.1016/s0142-9612(00)00423-3

[24] Naso F , Iop L , Spina M , Gerosa G. Are FDA and CE sacrificing safety for a faster commercialization of xenogeneic tissue devices? Unavoidable need for legislation in decellularized tissue manufacturing. Tissue Antigens 2014;83:193–4. 24397352 10.1111/tan.12275

